# Highly sensitive avoidance plays a key role in sensory adaptation to deep-sea hydrothermal vent environments

**DOI:** 10.1371/journal.pone.0189902

**Published:** 2018-01-03

**Authors:** Tetsuya Ogino, Shingo Maegawa, Shuichi Shigeno, Katsunori Fujikura, Haruhiko Toyohara

**Affiliations:** 1 Divison of Applied Biosciences, Graduate School of Agriculture, Kyoto University, Kyoto, Kyoto, Japan; 2 Intelligence Science and Technology, Graduate School of Informatics, Kyoto University, Kyoto, Kyoto, Japan; 3 Department for Marine Biodiversity Research, Japan Agency for Marine-Earth Science and Technology, Yokosuka, Kanagawa, Japan; 4 Biology and Evolution of Marine Organisms, Stazione Zoologica Anton Dohrn, Villa Comunale, Naples, Italy; National Taiwan Ocean University, TAIWAN

## Abstract

The environments around deep-sea hydrothermal vents are very harsh conditions for organisms due to the possibility of exposure to highly toxic compounds and extremely hot venting there. Despite such extreme environments, some indigenous species have thrived there. Alvinellid worms (Annelida) are among the organisms best adapted to high-temperature and oxidatively stressful venting regions. Although intensive studies of the adaptation of these worms to the environments of hydrothermal vents have been made, little is known about the worms’ sensory adaptation to the severe chemical conditions there. To examine the sensitivity of the vent-endemic worm *Paralvinella hessleri* to low pH and oxidative stress, we determined the concentration of acetic acid and hydrogen peroxide that induced avoidance behavior of this worm, and compared these concentrations to those obtained for related species inhabiting intertidal zones, *Thelepus* sp. The concentrations of the chemicals that induced avoidance behavior of *P*. *hessleri* were 10–100 times lower than those for *Thelepus* sp. To identify the receptors for these chemicals, chemical avoidance tests were performed with the addition of ruthenium red, a blocker of transient receptor potential (TRP) channels. This treatment suppressed the chemical avoidance behavior of *P*. *hessleri*, which suggests that TRP channels are involved in the chemical avoidance behavior of this species. Our results revealed for the first time hypersensitive detection systems for acid and for oxidative stress in the vent-endemic worm *P*. *hessleri*, possibly mediated by TRP channels, suggesting that such sensory systems may have facilitated the adaptation of this organism to harsh vent environments.

## Background

The environments around deep-sea hydrothermal vents are very harsh for organisms. The vents spout seawater heated by the mantle at temperatures that often reach more than one hundred degrees Centigrade. Moreover, this water is typically acidic, and contains toxic compounds such as heavy metals, hydrogen sulfide and reactive oxygen species [1, 2]. Precipitates of minerals transported by the hydrothermal fluid can form large edifices called chimneys. A large number of invertebrates live on the chimney walls, where they depend on chemosynthetic microbes as a food source or as symbionts [3].

The Annelida worms Alvinellidae occupy niches near hydrothermal vents. The family Alvinellidae is categorized into two genera, *Alvinella* and *Paralvinella*. They are mainly found in tubes on chimney walls near the blowout ports of vents [4]. Recently, improvements of study methods and tools have made it possible to investigate the physico-chemical conditions of their habitats [5]. These investigations have revealed that the physico-chemical conditions of these habitats are highly fluctuating. *In situ* measurements at an Alvinellidae colony revealed that pH there ranged from 4.4 to 7.5, and temperature from 10 to 90°C [6]. Another survey showed that the temperature inside the tubes on the chimney fluctuated from 28.6°C to 84.0°C, while that above the tube openings ranged from 7.5°C to 40.0°C [7]. Thus, dealing with the fluctuating physico-chemical conditions in their habitats is key to the survival of Alvinellidae species ("alvinellids"). Several studies have demonstrated behaviors and molecular defenses that contribute to the remarkable thermal tolerance of alvinellids [8, 9, 10, 11]. However, little is known about how these worms tolerate the harsh chemical conditions in their environment.

Animals respond to toxic compounds after detecting them with specialized sensors. Transient receptor potential (TRP) channels are known to be molecular sensors used for such detection by animals ranging from nematodes to mammals [12, 13, 14]. TRPs have been reported to detect various stimuli such as acids, temperature and reactive oxygen species [15, 16, 17]. In the environment of hydrothermal vents, many other stimuli exist in addition to these stimuli. For instance, hydrogen sulfide is found there, and is also an activator of TRP channels [18]. These facts suggest that alvinellids may utilize TRP channels for sensing the surrounding chemical environment.

In the present study, to reveal the characteristic features of chemical detection in an alvinellid, *Paralvinella hessleri*, we examined the response of *P*. *hessleri* to two chemicals, acetic acid and hydrogen peroxide. The low pH of hydrothermal fluid was mainly due to carbonic acid, but hydrochloric acid was also an acid source at some types of hydrothermal vents [19, 20]. We attempted to examine the effect of these acids on the behavior of *P*. *hessleri*, but it was difficult to prepare acid solutions containing precise concentrations of these acid components because of their volatile nature in addition to the difficulty of handling hydrochloric acid on board research vessels due to its corrosiveness. We assumed that an H^+^ sensor involved in acid perception in *P*. *hessleri* could probably respond to various acids, because the sensor would probably recognize H^+^ irrespective of the type of acid. Acetic acid was therefore used as a representative acid because acetic acid is often used in nociception tests in crustaceans and mice [21, 22]. Hydrogen peroxide was used as a representative reactive oxygen species because it was found at hydrothermal vents. The responses of *P*. *hessleri* to acetic acid and hydrogen peroxide were compared with those of a related species, *Thelepus* sp., living in the intertidal zone. Finally, we used ruthenium red (RR), a nonselective blocker of TRP channels, to test the involvement of TRP channels in these responses [23, 24]. The findings of this study revealed that *P*. *hessleri* was highly sensitive to acetic acid and hydrogen peroxide, and TRP channels contributed to the detection of these chemicals.

## Materials and methods

### Animal collection

Field sampling areas were not protected areas. The study did not involve collecting any endangered or protected species. *Paralvinella hessleri* were collected onboard the research vessel “Natsushima” during research cruise NT12-10 (31°53.05′ N, 139°58.10′ E, 907 m depth, off Myojin-sho submarine caldera, onboard ID 1374–9; or 32°06.21′ N, 139°52.05′ E, 1294 m depth, off Myojin Knoll, onboard ID 1377–4), in the Izu-Ogasawara Arc, Japan. Samples were collected on the 25th or 29th of April 2012 with a remotely operated Hyper-Dolphin 3000 vehicle (HPD#1374 or #1377). A suction sampler was used for collection of *P*. *hessleri* residing on vent chimneys. The live animals were kept in non-aerated cold deep-sea water collected with the animals. *Thelepus* sp. was collected at the intertidal zone in Osaka prefecture of Japan on the 14th of June 2014. The genus of *Thelepus* sp. was identified according to Fauchald [25].

### Chemical avoidance tests

#### Chemical avoidance test of *Paralvinella hessleri*

**Fig 1:**
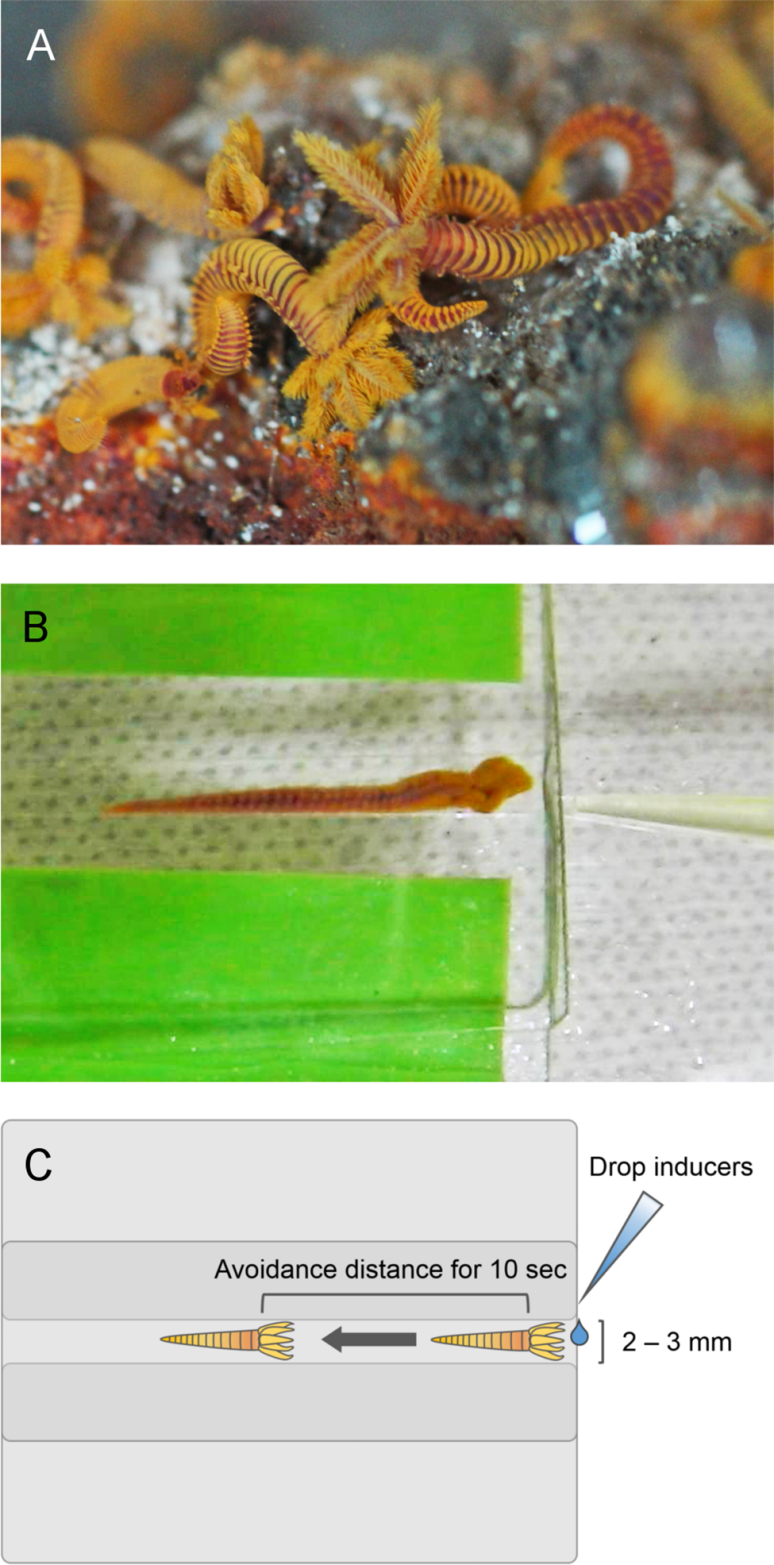
Chemical avoidance test of *Paralvinella hessleri*. (**A**) Live worms together with the chimney wall they inhabited viewed onboard the research vessel after collection. (**B**) A snapshot of the chemical avoidance test of *Paralvinella hessleri*. (**C**) A schematic drawing of the chemical avoidance test. A worm was inserted into an artificial tube made of three glass slides imitating a worm’s mucous tube. Inducers were dropped at the opening of the tube using a pipette.

Two glass slides were arranged in parallel with a space of 2–3 millimeters between them. Then another glass slide was put on top of these two slides to cover the space between them, thus creating a tube-like structure imitating the tube that these worms normally inhabit. One individual *P*. *hessleri* was inserted into “the tube” using its spontaneous backward movement. Ten microliters of inducer (0.01–1% acetic acid, or 0.003–0.3% hydrogen peroxide) were dropped at the opening of the tube (Fig 1; S1 Video). The chemical avoidance behavior was then recorded for 1 min after this induction using a video camera (Panasonic, HX-WA10). Ruthenium red treatment was performed as follows. *P*. *hessleri* was submerged in deep-sea water containing 1 mM RR for more than 10 min. Then, the treated worm was moved to a tube for the chemical avoidance test, which was performed as described above.

#### Chemical avoidance test of *Thelepus* sp

*Thelepus* sp. was placed on a plastic Petri dish (EIKEN CHEMICAL, AU3100), and artificial seawater (REI-SEA) was added to such an extent that the body of the *Thelepus* sp. was partially, but not completely, submerged in seawater. This experimental design was adopted in order to expose the worms directly to inducers. After at least 1 min, recording using a video camera (Panasonic, HX-WA30) was started. The worm's behavior without any treatment was recorded for more than 30 sec, and then 10 μL of inducer (0.01–10% acetic acid or 0.03–30% hydrogen peroxide) was dropped on the worm’s head (S2 Video), and its behavior was video-recorded for 1 min. The design of this assay and that performed on *P*. *hessleri* differed because of the long body size and long tentacles of *Thelepus* sp. For RR treatment, *Thelepus* sp. was submerged in sea water containing 1 mM RR for more than 10 min. Then the treated worm was moved to a Petri dish and subjected to the chemical avoidance test.

#### Image analysis

The video images recording the chemical avoidance test of *P*. *hessleri* were converted from mp4 into AVI format. The coordinates of the head at the start point and 10 sec after induction were determined using DippMotionPro 2D software (DITECT). The migration distance of each of these head coordinates was calculated as the distance between these two points. The video images of *Thelepus* sp. were also converted from mp4 into AVI format. The coordinates of the head were determined every half-second during the recording using DippMotionPro 2D software. Their migration distances every half-second were calculated using the coordinates' data obtained from the software. The post-induction migration distance minus the pre-induction migration distance was defined as the "avoidance index". The data without RR treatment were analyzed statistically using GraphPad Prism (MDF) by performing one-way ANOVA followed by Dunnett’s multiple comparisons (*P* < 0.1). The statistical significance of the difference between the results obtained with and without RR treatment was determined using Student’s *t*-test (*P* < 0.1).

## Results

### Chemical avoidance test of *Paralvinella hessleri*

To determine the concentrations of acetic acid and hydrogen peroxide that induced chemical avoidance behavior of *Paralvinella hessleri*, we conducted chemical avoidance tests. These tests revealed that the avoidance distances of the worm at 0.1% and 1% acetic acid were significantly longer than that for water, while that at 0.01% acetic acid was not (Table 1). The avoidance distances of the worm at 0.03% and 0.3% hydrogen peroxide were significantly longer than that for water, while that at 0.003% was not (Table 2). We then used RR treatment to examine whether TRP channels were involved in the avoidance behavior toward these chemicals. We found that RR treatment significantly decreased the avoidance distance induced by 0.1% acetic acid and 0.03% hydrogen peroxide (*P* < 0.1) (Tables 1 and 2).

**Table 1:** Chemical avoidance test of *Paralvinella hessleri* against acetic acid.

	- RR treatment		+ RR treatment	
Concentration (%)	Number of samples	Avoidance distance (mm)	Number of samples	Avoidance distance (mm)
0	3	3.19 ± 2.21	ND	ND
0.01	3	0.66 ± 0.66	ND	ND
0.1	3	24.51 ± 12.97*	3	4.93 ± 3.11^†^
1	3	26.75 ± 6.62*	3	19.81 ± 14.94

Values are mean ± SD; ND: not determined.

An asterisk (*) indicates a significant difference between the avoidance distance in the "0" control condition without acetic acid and the avoidance distance with addition of the indicated concentration of acetic acid, as determined by one-way ANOVA with Dunnett's post hoc test (*P* < 0.1).

A dagger (^†^) indicates a significant difference between the avoidance distance with ruthenium red treatment as compared to that without ruthenium red treatment, as determined by Student's *t*-test (*P* < 0.1).

**Table 2:** Chemical avoidance test of *Paralvinella hessleri* against hydrogen peroxide.

	- RR treatment		+ RR treatment	
Concentration (%)	Number of samples	Avoidance distance (mm)	Number of samples	Avoidance distance (mm)
0	3	3.19 ± 2.21	ND	ND
0.003	3	1.99 ± 0.40	ND	ND
0.03	3	18.16 ± 8.05*	3	3.84 ± 3.51^†^
0.3	3	28.07 ± 10.60*	6	22.16 ± 12.07

Values are mean ± SD; ND: not determined.

An asterisk (*) indicates a significant difference between the avoidance distance in the "0" control condition without hydrogen peroxide and the avoidance distance with addition of the indicated concentration of hydrogen peroxide, as determined by one-way ANOVA with Dunnett's post hoc test (*P* < 0.1).

A dagger (^†^) indicates a significant difference between the avoidance distance with ruthenium red treatment as compared to that without ruthenium red treatment, as determined by Student's *t*-test (*P* < 0.1).

### Chemical avoidance test of *Thelepus* sp.

To compare the sensitivity of *P*. *hessleri* to chemicals with the sensitivity of related species inhabiting the intertidal zone, we conducted chemical avoidance tests on *Thelepus* sp. At first, we tried to adapt the method we had used for *P*. *hessleri*, but this method did not work properly due to the larger body size and longer tentacles of this species. To avoid the difficulty of using the tube-like structure test to evaluate the chemical avoidance behavior of *Thelepus* sp., we instead measured the movement of the head to evaluate the effects of various chemicals. In this test, we made an effort to drop the chemicals directly on the head in order to apply the same inducing stimulus as that applied in the test for *P*. *hessleri* as far as possible. The avoidance index of this worm against 10% acetic acid was significantly higher than that against water, while acetic acid at lower concentrations did not affect the avoidance behavior compared to that against water (Table 3). The avoidance index of the worm at 30% hydrogen peroxide was significantly higher than that against water, while hydrogen peroxide at lower concentrations did not cause a higher avoidance index compared to water (Table 4). To test the possible involvement of TRP channels in the avoidance behavior, we examined the avoidance index of *Thelepus* sp. after RR treatment. RR treatment did not decrease the avoidance index at any concentration of acetic acid tested, but increased the avoidance index at 0.1% acetic acid (*P* < 0.1, Table 3). RR treatment significantly decreased the avoidance index at 30% hydrogen peroxide (*P* < 0.1, Table 4).

**Table 3:** Chemical avoidance test of *Thelepus* sp. against acetic acid.

	- RR treatment		+ RR treatment	
Concentration (%)	Number of samples	Avoidance index (mm)	Number of samples	Avoidance index (mm)
0	5	4.96 ± 5.09	5	3.75 ± 6.16
0.01	5	-0.54 ± 6.13	ND	ND
0.1	5	-1.87 ± 1.36	4	6.21 ± 1.74^†^
1	5	10.93 ± 10.30	5	18.41 ± 7.02
10	5	28.22 ± 20.94*	5	29.63 ± 16.14

Avoidance index was calculated as the post-induction migration distance during 30 sec minus the pre-induction migration distance during 30 sec. Values are mean ± SD; ND: not determined.

An asterisk (*) indicates a significant difference between the avoidance index in the "0" control condition without acetic acid and the avoidance index at the indicated concentration of acetic acid without RR treatment, as determined by one-way ANOVA with Dunnett's post hoc test (*P* < 0.1).

A dagger (^†^) indicates a significant difference between the avoidance index with ruthenium red treatment as compared to that without ruthenium red treatment, as determined by Student's *t*-test (*P* < 0.1).

**Table 4:** Chemical avoidance test of *Thelepus* sp. against hydrogen peroxide.

	- RR treatment		+ RR treatment	
Concentration (%)	Number of samples	Avoidance index (mm)	Number of samples	Avoidance index (mm)
0	5	4.96 ± 5.09	5	3.75 ± 6.16
0.03	4	-5.23 ± 6.16	ND	ND
0.3	5	6.98 ± 5.25	5	2.41 ± 2.89
3	7	11.52 ± 9.08	5	19.50 ± 4.57
30	4	32.46 ± 19.20*	4	6.86 ± 13.07^†^

Avoidance index was calculated as the post-induction migration distance travelled during 30 sec minus the pre-induction migration distance travelled during 30 sec. Values are mean ± SD; ND: not determined.

An asterisk (*) indicates a significant difference between the avoidance index in the "0" control condition without hydrogen peroxide and the avoidance index with the indicated concentration of hydrogen peroxide without RR treatment, as determined by one-way ANOVA with Dunnett's post hoc test (*P* < 0.1).

A dagger (^†^) indicates a significant difference between the avoidance index with ruthenium red treatment as compared to that without ruthenium red treatment, as determined by Student's *t*-test (*P* < 0.1).

## Discussion

In our chemical avoidance test against acetic acid, *Paralvinella hessleri* showed significantly greater avoidance distance at concentrations of 0.1% (pH 3.2) and 1% (pH 2.7) acetic acid (Table 1). The hydrothermal fluids released from hydrothermal vents are acidic (around pH 3), while the surrounding water has a stable, slightly alkaline pH (pH 7.8) [26]. These features cause a steep gradient of proton concentration at the boundary between the hydrothermal fluids and surrounding water, namely, at the habitat of *P*. *hessleri*. Environmental acids cause toxic effects in aquatic animals by disturbing ionic or/and osmotic regulation [27, 28]. Therefore, it seems reasonable to expect that *P*. *hessleri* might monitor environmental protons in order to detect the approach of hazardous hydrothermal fluids. In contrast, the pH of the water which *Thelepus* sp. inhabits is stable and slightly alkaline. Therefore, it is not necessary for *Thelepus* sp. to respond to environmental pH. This is in agreement with our finding that *Thelepus* sp. did not display avoidance behavior over the physiological pH range (Table 3).

In the chemical avoidance test against hydrogen peroxide, *P*. *hessleri* showed significantly increased avoidance distances at 0.03% and 0.3% hydrogen peroxide (Table 2). Hydrothermal fluid itself contains no oxygen or reactive oxygen species (ROS) such as hydrogen peroxide [26]. However, ROS can be produced when both sulfide and molecular oxygen are present in the surrounding water, suggesting that ROS might be produced at the boundary between hydrothermal fluids and surrounding water [29]. In fact, hydrogen peroxide has been reported to exist in the environment of hydrothermal vents [2]. Because ROS, including hydrogen peroxide, can damage biomolecules [30], it is advantageous for organisms such as *P*. *hessleri* to be able to sense hydrogen peroxide in order to avoid exposure to it. In the intertidal zone, hydrogen peroxide is produced photochemically, and ranges in concentration from low nM levels to 440 nM in surface seawater. [31]. *Thelepus* sp. showed avoidance behavior only toward 30% hydrogen peroxide (Table 4). Hydrogen peroxide is not known to be present at such a high concentration in the natural environment of *Thelepus* sp., and hence it is not necessary for *Thelepus* sp. to respond behaviorally to environmental ROS.

Behavioral responses of aquatic animals to environmental acids have been reported so far for fishes [32, 33], crustaceans [34, 35] and mollusks [36, 37]. The contribution of TRPs to behavioral responses toward low pH was shown in nematode [15], suggesting the possible involvement of TRPs in the behavioral response to acids in other aquatic animals as well. In contrast, behavioral responses of aquatic animals toward environmental ROS have never been reported. However, TRPs were shown to be involved in the detection of hydrogen peroxide in mouse [38], suggesting the possibility that TRPs might be involved in the behavioral response to hydrogen peroxide in aquatic animals. In the chemical avoidance test in the present study, *P*. *hessleri* and *Thelepus* sp. responded to acids and hydrogen peroxide. To test the involvement of TRPs in the behavioral response to acids and hydrogen peroxide, chemical avoidance tests were conducted after the administration of a non-selective TRP channel blocker, ruthenium red.

Ruthenium red treatment significantly decreased the avoidance length of *P*. *hessleri* against 0.1% acetic acid and 0.03% hydrogen peroxide but not against higher concentrations (Tables 1 and 2), indicating that TRP channels mediated the detection by *P*. *hessleri* of toxic chemicals at low, but not high, concentrations. These results imply that receptors other rather than TRP channels are involved in the detection of acetic acid and hydrogen peroxide at such higher concentrations. TRPs and ryanodine receptors (RyRs) are Ca^2+^ channel proteins that may be involved in animal behavior, and RR is known to be an antagonist against these Ca^2+^ channels [39, 40] RyR functions to release Ca^2+^ from the sarcoplasmic reticulum and to induce muscle contraction [41], suggesting that muscle contraction would be suppressed in an animal if RR inhibited the animal’s Ca^2+^ transport through RyR. In the present study, however, RR showed no inhibitory effect on the body movement associated with muscle contraction on either species examined (Tables 1–4), which suggests that RR does not have an antagonistic effect against RyR of either species. This might be because the rate of permeation of RR into the animals was so slow [40] that RR did not reach RyR in muscle and only reacted with Ca^2+^ channels localized in the skin during the experimental period.

The TRP channel family consists of many members and is divided into several subfamilies [23]. In mammals, TRPV1, TRPA1 and some other members of the TRP channel family have been reported to be activated by protons [42, 43]. *Caenorhabditis elegans* shows avoidance behavior against acidic pH (pH 3–5), which is detected in this animal by OSM-9, a member of the TRPV subfamily [15]. Interestingly, this pH is close to that which induces avoidance behavior of *P*. *hessleri*, which implies that similar TRP channels may be involved in the acid avoidance of *P*. *hessleri*. Reactive oxygen species, including hydrogen peroxide, are detected by mammals via TRPA1 [17, 38], TRPM2 [44] and some other members of the TRP family. We speculate that homologue of these TRPs are possible candidates for the hydrogen peroxide sensor of *P*. *hessleri*. Further studies will be needed to identify the particular TRP channels that act as chemical sensors in *P*. *hessleri* and to clarify their functions.

## Conclusions

Our newly developed assay reported here can easily evaluate the avoidance behavior of a deep-sea hydrothermal vent animal, *P*. *hessleri*, onboard a research vessel. Our findings here using this assay revealed that *P*. *hessleri* showed avoidance behavior against two environmentally relevant inducers with high sensitivity. Its ability to escape from toxic chemicals even at low concentrations should contribute to its ability to occupy a niche in the vicinity of hydrothermal vents. In addition, our results suggested that TRP channels act as sensors of these chemicals. In future studies, we will attempt to identify the particular TRP channels that act as chemical sensors and clarify their functions in hydrothermal vent animals, which will lead to unraveling the chemical sensing mechanisms of these animals.

## Supporting information

S1 Video Chemical avoidance test on *Paralvinella hessleri*. A worm was inserted into an artificial tube made of three glass slides. Then an inducer was dropped at the opening of the tube and the behavior was recorded using a video camera (Panasonic, HX-WA10). The inducer used in this video was 0.1% acetic acid.
(MP4)

S2 Video Chemical avoidance test on *Thelepus* species. A worm was put in a Petri dish with artificial sea water. Then an inducer was dropped at the head of the worm and the behavior was recorded using a video camera (Panasonic, HX-WA30). The inducer used in this video was 10% acetic acid.
(MP4)
